# Endometrial Mixed and Mixed-Feature Carcinomas: Small Cohort Clinicopathologic and Molecular Studies

**DOI:** 10.3390/cancers18030440

**Published:** 2026-01-29

**Authors:** Swati Bhardwaj, Mona Saleh, Yayoi Kinoshita, Rachel Brody, Olga Lukatskaya, Stephanie V. Blank, Brett Baskovich, Tamara Kalir

**Affiliations:** 1 Department of Pathology, Molecular and Cell Based Medicine, Icahn School of Medicine at Mount Sinai, New York, NY 10029, USAyayoi.kinoshita@mountsinai.org (Y.K.); rachel.brody@mountsinai.org (R.B.); bbaskovich@sonichealthcareusathyroseq.com (B.B.); 2 Department of Obstetrics, Gynecology and Reproductive Science, Icahn School of Medicine at Mount Sinai, New York, NY 10029, USAstephanie.blank@mountsinai.org (S.V.B.); 3 Department of Molecular Pathology, Icahn School of Medicine at Mount Sinai, New York, NY 10029, USA; olga.lukatskaya@mountsinai.org

**Keywords:** mixed endometrial cancer, mixed-feature endometrial cancer, molecular genetics, endometrial cancer

## Abstract

Objectives: To examine the clinical, pathologic, and molecular features of mixed and mixed feature endometrial carcinomas and compare them to pure serous carcinoma and pure endometrioid carcinoma. Methods: The study analyzed the clinical characteristics, histologic composition, and molecular genetic profiles of mixed and mixed feature endometrial cancers, with a focus on shared and distinct mutations. Patient demographics, disease-free survival, and molecular alterations, including in *TP53*, *PIK3CA*, *TERT*, *MAP2K1* genes, and *ERBB2* gene amplifications, were assessed and compared to pure serous and pure endometrioid carcinomas. Results: Patients with mixed and mixed-feature carcinomas were older (median age: 73 years) and had worse disease-free survival (median: 23 months) than those with pure endometrioid carcinoma (median: 48 months). Mixed and mixed-feature carcinomas were histologically high-grade, most commonly comprising serous and endometrioid components. Molecular profiling supported a clonal origin of these tumors, with identical *TP53* and *PIK3CA* gene mutations between the two histologic components in each case. There were additional gene mutations (e.g., *TERT* and *MAP2K1*) found in higher-grade components. *ERBB2* amplifications were more frequent in the mixed carcinomas groups (33%) compared to pure serous (11%) and pure endometrioid carcinomas (0%). Some of the mixed and mixed-feature carcinomas also showed *FBXW7* mutations, not seen in either the pure endometrioid or pure serous carcinomas. Conclusions: Mixed and mixed-feature carcinomas share origins with pure endometrial serous and endometrioid carcinoma subtypes but exhibit distinct molecular alterations. These findings highlight the importance of molecular subtyping for diagnosis and treatment planning. Future research could focus on larger cohorts and targeted sequencing to better understand the pathogenesis of mixed and mixed-feature carcinomas in order to refine therapeutic strategies.

## Simple Summary

Endometrial cancer is a prevalent disease worldwide. There are different kinds of endometrial cancers and their treatment is based on the specific type of cancer—termed the histologic type, and the extent of disease spread—termed stage. The pathology diagnosis of the specific type of endometrial cancer is improving because of our ability to identify specific gene mutations that are unique to the different histologic groups of endometrial cancer. Discovering more about these gene mutations will enable us to design better, more personalized treatment, and avoid having patients try medicines that may not be effective at eliminating their tumor cells. In this current research investigation, we studied a rare sub group of endometrial cancers called mixed carcinomas. There are currently no treatment guidelines for this particular group, and we wanted to learn more about their gene mutations in order to better guide future therapy.

## 1. Introduction

In the western world, endometrial cancer comprises the majority of uterine corpus malignancies, with an increasing incidence in developing countries [1,2,3,4,5]. Historically, Bokhman’s proposed [6] two-tiered classification of endometrial cancers, estrogen-related and non-estrogen-related, had been utilized; with the estrogen-related tumors mostly having a better prognosis and the non-estrogen-related tumors generally exhibiting a more aggressive behavior. However, the histologic diagnosis of high-grade endometrial adenocarcinomas, both estrogen-related and non-estrogen-related has been shown to present challenges to pathologists; in many cases with moderate to poor diagnostic concordance among experts [7,8,9,10]. Given that histologic diagnosis along with disease stage are key cornerstones in disease management [11], the time was ripe for improvement. In 2013 an important development addressed this issue. The Cancer Genome Atlas Research Network published the identification of four distinct molecular groups of endometrial cancers [12], described as: (1) POLE (polymerase epsilon) exonuclease domain ultramutated, (2) microsatellite instability hypermutated, DNA mismatch repair deficient, (3) TP53 gene mutated (copy number high), and (4) tumors lacking a specific mutational profile (copy number low) [11,13]. This new molecular classification led to the currently integrated molecular and histologic classification of endometrial cancers, has improved inter-observer concordance, and offers better potential for patient stratification and treatment [14,15]. Following this, the International Federation of Gynecology and Obstetrics (FIGO) has included features of the molecular classification, such as *TP53* and POLE mutation status as important prognostic information, in their updated 2023 staging classification [16,17,18]. Clinical trials are currently ongoing and treatment recommendations are aimed at incorporating molecular subtyping data along with the histologic diagnosis [17,19,20,21,22]. Although our understanding of endometrial cancer has improved, there is still much to learn, as more recent studies have noted both clinical and molecular heterogeneity within the four different classes of the molecular subtypes [23], and the rarer histotypes are still not well characterized. Toward this end, we ventured to study the so-called mixed endometrial carcinomas.

Mixed endometrial carcinoma is defined by the World Health Organization (WHO) as a carcinoma composed of two or more discrete histologic types of endometrial carcinomas, at least one of which is a high-grade component; either a clear cell or serous carcinoma [24], and there is no minimal amount necessary to qualify as a distinct component. Mixed carcinomas are a rare subset of endometrial tumors, with the mixed endometrioid and serous combination being the most common histologic type, accounting for roughly 10% or less of endometrial carcinomas [25]. Mixed carcinomas are considered high-grade regardless of the relative percentage of the serous or clear cell component, and have been included among the other high-grade carcinomas in many studies. Owing to their rarity, there is a paucity of data pertaining to their biology and clinical behavior. As a peculiar histologic subtype, the pathogenesis of mixed carcinomas has been intriguing from both scientific and clinical aspects. In one of the earliest and largest studies on the molecular origins of mixed carcinomas, Kobel [26] found a clonal origin in approximately 90% of their study cohort of 18 cases, concluding morphologic mimicry, and less commonly, early molecular divergence as the proposed mechanism of origin. Similar clonal origins have been established by others [27] while a biclonal origin has been suggested by some [28]. The molecular underpinnings of mixed carcinomas are even more relevant in the context of the updated FIGO classification [17].

The goal of the current study was to further characterize the clinical and pathologic features, clinical behavior, and molecular genetic profiles of mixed endometrial carcinomas, compared to pure serous carcinomas and pure endometrioid carcinomas.

## 2. Materials and Methods

Study approval was obtained by the Icahn School of Medicine at Mount Sinai Institutional Review Board (IRB) (STUDY-21-00951). A waiver of informed consent was granted as a number of patients were no longer under follow-up and some patients were deceased at the time of the study. To enable several years of clinical follow-up, the pathology database from 2016 to 2018 was searched to retrieve cases with a pathologic diagnosis in the clinical setting of mixed carcinoma; cases of pure serous and pure endometrioid carcinomas were also retrieved as controls. Given that mixed carcinomas are rare, we were able to find only nine cases during this time period. The WHO definition had been updated during the time of our study and while four of our nine cases qualified for mixed carcinoma given they exhibited two spatially distinct components, with three cases showing a serous/endometrioid combination and one case a clear cell/endometrioid combination, it was seen upon review that five of the nine cases were difficult to classify due to exhibiting a mixed or intermingled phenotype which appeared somewhat serous and somewhat endometrioid without either of these features being spatially distinct. Due to the paucity of our cases and not having another category in which to place these five cases, we decided to include these tumors in our study of mixed groups as so-called mixed-feature carcinomas. Cases were reviewed to obtain relevant pathologic information. Clinical and demographic data were extracted from the medical records.

### 2.1. Immunohistochemistry

Immunohistochemistry (IHC) was performed to aid and confirm diagnosis. Where possible, p16 and p53 IHC were performed on both morphologically distinct elements of the tumor. DNA mismatch repair (MMR) IHC was performed in selected cases that had been requested by clinicians, as this study included cases prior to 2017—when universal testing for MMR proteins became standard of care. Tumor blocks were cut at 5-µm thickness, then deparaffinized, and subjected to antigen retrieval using 10 mM citrate buffer at 92 °C for 30 min. Immunohistochemistry was performed on all cases using a monoclonal antibody for p53. P53 staining was reported as positive (mutation type) if there was an overexpression of p53 (greater than 80% of tumor cells showing strong or 3+ nuclear staining intensity), or a null-type pattern was observed. None of our cases showed a cytoplasmic staining pattern [21,29].

### 2.2. Molecular Analysis

Serous, endometrioid, and separately demarcated and sampled distinct components of mixed carcinomas were sent for targeted sequencing. In four of the mixed carcinoma cases, the two distinct components could be separately isolated and were analyzed separately. A distinct isolation of the two components was not possible in the five mixed feature cases- these were analyzed as a whole (including both endometrioid and higher-grade intermingled components). A DNA library was prepared using the Oncomine Comprehensive Assay v3M (Thermo Fisher Scientific A36111, Waltham, MA, USA). Twenty (20) ng of extracted DNA from each sample was used for input for DNA library prep using the Oncomine Comprehensive Assay v3M (Thermo Fisher Scientific A36111). Final libraries were quantified by qPCR using the Ion Library Taqman Quantitation Kit (Thermo Fisher Scientific 4468802) and sequenced on the Ion S5 XL System (Thermo Fisher Scientific A27214) with templating performed on the Ion Chef Instrument (Thermo Fisher Scientific 4484177). All samples were sequenced using the Ion 550 Chip Kit (Thermo Fisher Scientific A34537) and Ion 550 Chef Kit (Thermo Fisher Scientific A34541). Base-calling as well as primary analysis and variant calling were performed using Torrent Software Suite (TSS 5.12.3).

## 3. Results

A total of 4 mixed endometrial carcinomas and 5 mixed-feature carcinomas (9 total) were identified from 2016–2018 and included in the study. For comparison of clinical and histologic findings, 9 pure serous carcinomas and 10 pure endometrioid carcinomas were also included in the same study period. All histology slides were re-reviewed to corroborate histologic features (Table 1).

Patients from the mixed endometrial carcinoma group were older; median age 73 years, compared to 64 years for pure endometrioid carcinoma and 68 years for pure serous carcinoma (Table 1). The average BMI in endometrioid carcinomas was 38, compared to 33 in serous carcinoma and 30 in the mixed carcinomas group (Table 1).

Follow-up information was available in 19 cases (6 serous, 8 mixed and 5 endometrioid carcinomas). Upon histologic review, the most common combination seen in mixed carcinomas was serous and endometrioid adenocarcinoma (8/9 cases) with only one case of mixed clear cell and endometrioid adenocarcinomas. The amount of serous carcinoma component ranged from <5% (case M1) to 90% (case M11). The only case with mixed clear cell and endometrioid adenocarcinomas showed 95% clear cell and 5% endometrioid histology.

The five cases of mixed-feature endometrial carcinoma were subjected to molecular sequencing entirely as the two components could not be reliably isolated separately due to intermingling of the two components (cases M3, 4, 6, 7, 8). Three out of these five cases showed oncogenic *TP53* mutations. Other alterations observed in these cases included mutations in *PIK3CA* (1/5), *PTEN* (1/5), *KRAS* (1/5), *FBXW7* (2/5), *SETD2* (1/5), *MTOR* (1/5), *RAD51B* (1/5) and *ERBB2* (1/5); and amplifications of *ERBB2* (2/5), *CCNE1* (1/5) and *AKT2* (1/5).

The four cases of mixed endometrial carcinoma were analyzed with separate molecular analysis of each individual component (cases M5, 9, 10, 11). All four cases showed at least one shared (identical) mutation between both the endometrioid and the serous/clear cell components. The most common shared mutations between these two components were in *TP53* (2/4) and *PIK3CA* (2/4). Other shared mutations included *PTEN* (1/4), *PPP2R1A* (1/4) and *KRAS* (1/4) mutations, *MYC* amplification (1/4) and *FBXW7* mutation (1/4). The *FBXW7* mutation was seen in one mixed and two mixed-feature carcinomas (total 3/9), and was not seen in any of the pure endometrioid or pure serous carcinomas. The higher-grade component in the mixed carcinomas showed additional mutations in genes including *TP53*, *TERT*, *MAP2K1*, *ARID1A*, *FANCI*, and *SMARCB1*. In case M11, no additional or unique mutation was seen in either of the components, with all genetic alterations being the same in both components. In cases M5 and M9, it was the higher-grade component (serous or clear cell) that showed additional mutations. In case M10, both components showed additional mutations in addition to the common/shared mutations.

**Figure 1:**
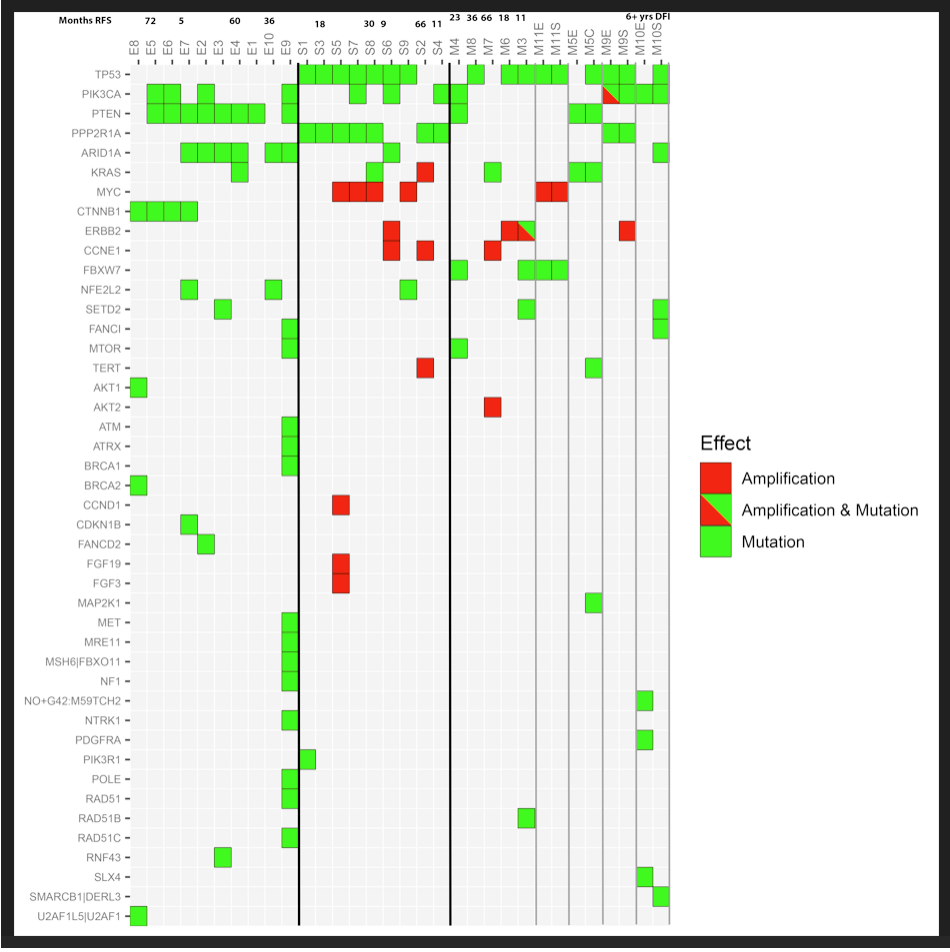
Oncoplot of mutations in pure and mixed endometrial carcinomas.

An Oncoplot (Figure 1) shows that the mixed carcinomas exhibited shared gene mutations with the pure endometrioid and the pure serous groups, while the pure groups exhibited mutations more distinct from each other. *PIK3CA* and *PTEN* mutations were most common in pure endometrioid carcinoma and the endometrioid component of mixed carcinoma. Similarly, *TP53* mutations were seen in pure serous carcinomas as well as the serous component of mixed carcinomas (Figure 1). Interestingly, *ERBB2* amplifications were most commonly seen in the mixed carcinomas (3/9 cases) as compared to serous carcinoma (1/9 cases) or endometrioid carcinoma (0/9 cases), (Figure 1).

Mutations in *TP53* occurred in both the pure serous carcinomas and the serous component of the mixed carcinomas (Table 2), and not in our pure endometrioid carcinomas, three of which were high-grade (previously shown in Table 1).

While we did not identify any *TP53* mutations that were unique to either group, we found the argenine to histidine substitution at site 175 in the protein (p.R175H, also written as p.Arg175His) to be noteworthy (Table 2). This mutation was seen in two pure serous carcinomas and in one mixed carcinoma. While all three tumors in our small cohorts with the p.Arg175His mutation were low stage (stage IB), they all exhibited lymphovascular space invasion and the one serous and one mixed case with follow up both had recurrences. The other serous case was lost to follow up.

## 4. Discussion

### 4.1. Summary of Main Findings

This study provides an in-depth analysis of the clinical, histologic, and molecular characteristics of a very small cohort of mixed endometrial carcinomas, a rare but clinically significant subtype of endometrial cancer. Our mixed carcinomas were found to occur predominantly in older patients, with a median age of 73 years; this observation has been reported by others [30]. Mixed carcinomas also appeared to have a worse disease-free survival (23 months) compared to endometrioid carcinomas (48 months). Because of our small case numbers, the disease free survival values were derived from each entire individual group and not adjusted for confounders such as stage, treatment and co-morbidity; and while statistical significance was not achieved, a similar finding has been reported by others [31]. Histologically, the majority of mixed carcinomas in this cohort comprised serous and endometrioid components, aligning with previous reports that mixed endometrioid and serous carcinoma is the most common subtype [25]. Molecular analysis provided further insight into mixed carcinoma pathogenesis, revealing identical gene mutations between the two components in all cases where separate sequencing was possible, suggesting a clonal origin in most cases. However, additional mutations found in the higher-grade component, including alterations in *TERT*, *MAP2K1*, and *ARID1A*, indicate a pattern of molecular evolution, potentially contributing to tumor progression. A key novel finding in this study was the higher frequency of *ERBB2* amplifications in mixed carcinomas (33%) compared to pure serous carcinoma (11%) and pure endometrioid carcinoma (0%), suggesting a distinct molecular feature that may have therapeutic implications. While our study includes a very small patient cohort, this finding of robust *ERBB2* amplification in mixed endometrial carcinomas has been corroborated in a recently published, larger cohort study [30].

Tumor suppressor *FBXW7* [32] was seen to be mutated in 1/3rd of our small mixed carcinoma group (1 mixed carcinoma and 2 mixed-feature carcinomas), and was not found to be mutated in either of our small pure serous or pure endometrioid carcinoma groups. The *FBXW7* gene mutation was seen to occur in cancers also harboring mutations in one or both genes *TP53* and *PIK3CA*, but was also seen to occur in the absence of these mutations. Of interest, it has been reported [33] that in genetically-engineered mouse models and human endometrial carcinoma cell lines, missense-mutated *FBXW7* protein alone does not cause endometrial neoplasia but, in apparent interplay with missense-mutated *TP53* or *PTEN* loss, seems to accelerate endometrial carcinogenesis and may provide, via the Wnt pathway another potential target for endometrial cancer therapy [33]. In our three cases with FBXW7 mutation, each of which was a missense mutation, one showed missense *TP53* mutation and one showed a nonsense (truncated) *TP53* mutation, and the other showed nonsense (truncated) *PTEN* mutation. The *FBXW7* gene may hold promise for further investigation [34].

While *TP53* gene mutations were varied among the carcinomas and did not show specificity to any group, the majority resulted in truncated and/or otherwise inactivated proteins [35]. However the p.Arg175His mutation was noteworthy. This mutation is common in various cancers and results in changes to DNA binding activity, resulting in loss of normal tumor suppressor functions as well as novel ‘gains of function’ which induce genetic instability and promote tumor growth [36]. Along this line, Behring et al. [37] found that breast cancers with *TP53* mutation in the p.Arg175His locus had a higher frequency of being immune-rich; with an increase in non-activated M0 macrophages and a decrease in activated M1 macrophages, resulting in decreased phagocytic capacity, enhanced extracellular matrix degradation, higher invasiveness and worse survival compared to all other *TP53* mutations. They postulated that this could involve a type of p53 protein gain-of-function through the immune response or possibly a transcriptional mechanism; as the mutation is located in the DNA-binding domain of the protein. If true and applicable in endometrial cancer, this finding could explain the tendency to recurrence in our low-stage cases, although this is conjectural and recurrence was not unique to this particular gene mutation.

Our findings highlight the complex nature of mixed endometrial cancers and endometrial cancer in general, and reinforce the need for more and detailed molecular studies, as these will provide important information for better prognostication and personalized, targeted therapy.

### 4.2. Results in the Context of Published Literature

The findings of this study align with previous research that has demonstrated a predominantly clonal origin in mixed endometrial carcinomas. One of the earliest studies by Kobel et al. [26] reported that 90% of the mixed endometrial carcinomas in their cohort exhibited a clonal relationship between the different histologic components, a conclusion supported by our data showing shared mutations in genes such as *TP53*, *PIK3CA*, and *PTEN* across components. This strengthens the argument that mixed endometrial carcinomas arise from a common progenitor cell rather than being the result of independent tumor clones arising within the same endometrium. The observation of shared molecular alterations aligns with the findings of other studies, which have shown that mixed endometrial carcinomas often originate from a single progenitor clone, with subsequent molecular evolution leading to distinct histologic subtypes [27]. However, this study also provides additional evidence of molecular divergence, with high-grade components acquiring additional mutations not seen in the lower-grade endometrioid component. This supports a model of early clonal divergence or tumor progression, where a common ancestor cell accumulates further genetic alterations that drive the histologic transformation into a higher-grade phenotype.

Interestingly, a minority of studies have proposed a biclonal origin in mixed endometrial carcinomas [28], suggesting that early molecular divergence may contribute to the formation of discrete histologic components, at least in some tumors. In our study however, biclonal origins were not evident, as all analyzed cases showed shared mutations. However, the lack of any additional mutations in either of the components in case M11 could potentially be an example of morphologic mimicry rather than divergent differentiation. These findings support the idea that mixed endometrial carcinomas may arise by differing mechanisms, the most common being origin from a single progenitor clone followed by divergent differentiation however, other mechanisms may be operative in other cases.

We also found a similarity between the molecular profiles of the serous and endometrioid components of mixed endometrial carcinomas and their pure serous and endometrioid carcinoma counterparts. This consistency reinforces the molecular classification framework proposed in the updated FIGO 2023 classification, which emphasizes the importance of molecular subtypes in endometrial carcinoma grading [17].

The role of *ERBB2* amplification in mixed endometrial carcinomas is a particularly novel finding. Interestingly, *ERBB2* amplifications were more frequently observed in our mixed carcinomas (3/9 cases) than in our pure serous carcinomas (1/9 cases) and our pure endometrioid carcinomas (0/9 cases). While *ERBB2* amplifications have been previously reported in serous carcinomas, the significantly higher frequency observed in mixed endometrial carcinomas suggests that this genetic alteration may be a distinguishing feature of this tumor type. This finding has been corroborated by a recently published, large cohort study [30] and suggests that mixed endometrial carcinomas may possess unique molecular alterations that differentiate them from their pure carcinoma counterparts. The role of ERBB2 amplification in mixed endometrial carcinoma pathogenesis and its potential as a therapeutic target warrants further investigation.

### 4.3. Strengths and Weaknesses

This study has several strengths that contribute to the understanding of mixed endometrial carcinomas. First, it provides a detailed molecular characterizations of these tumors, using targeted sequencing to compare molecular alterations across histologic components within the same tumor. This approach allows for a nuanced understanding of tumor evolution and the potential drivers of high-grade transformation in mixed endometrial cancers. Another key strength is the inclusion of a comparison cohort of pure endometrioid and serous carcinomas, enabling direct evaluation of how mixed carcinomas relate to their morphologically similar counterparts. The study also adheres to strict WHO-defined diagnostic criteria for mixed carcinoma classification, reducing the risk of misclassification and ensuring that findings are applicable to clinical practice.

Despite these strengths, there are also important limitations. The study cohort is relatively small, reflecting the rarity of mixed carcinomas, which can limit the generalizability. Additionally, in five cases, the two histologic components were too intermingled to allow for separate sequencing, meaning that molecular divergence in these tumors could not be fully assessed. This highlights a separate area in need of further investigation, as these tumors were not clearly of the endometrioid or non-endometrioid groups. This limitation highlights a challenge in tumors in which the histologic features are not clearly of endometrioid or non-endometrioid subtype, and the immunophenotype is also uncharacteristic of either group. Nonetheless, the findings in both the mixed and mixed feature carcinomas were more similar to each other in terms of patient age, disease free survival, *ERBB2* amplification and *FBXW7* mutation, as compared to the pure serous and pure endometrioid carcinoma groups, suggesting that the WHO definition for mixed carcinomas may exclude some cases. Another limitation is the lack of mismatch repair data on all cases. The retrospective nature of the study and reliance on archived tissue samples may also introduce selection bias. Furthermore, the lack of functional validation of molecular findings—such as assessing the biological impact of *ERBB2* amplification—means that the potential therapeutic implications remain speculative at this stage. Future studies may seek to confirm these findings in larger, multi-institutional cohorts and explore the functional relevance of key molecular alterations identified in mixed endometrial carcinomas.

### 4.4. Implications for Practice and Future Research

The findings from this study may have important implications for both clinical practice and future research. The identification of frequent *ERBB2* amplifications in mixed endometrial carcinomas suggests that this gene alteration may serve as a predictive and prognostic marker in chemo- and radiation therapies of mixed endometrial cancers. HER2-targeted therapies have shown efficacy in serous endometrial carcinomas, and may also be beneficial for patients with mixed endometrial carcinomas. This is particularly relevant in light of the updated 2023 FIGO classification, which now incorporates molecular subtypes into endometrial cancer staging. Routine molecular subtyping may refine risk stratification and therapeutic decision-making, ensuring that patients with high-risk molecular alterations receive appropriate targeted therapies.

Regarding targeted therapy, future research may focus on whole-genome sequencing and transcriptomic analysis, which could provide deeper insights into the molecular pathways driving tumor progression. Identifying cellular pathways impacted by co-mutations such as tumor suppressor genes *FBXW7* and *TP53* may enhance management of endometrial carcinomas.

## 5. Conclusions

This study adds to our understanding of mixed endometrial carcinomas by demonstrating a shared clonal origin, with additional mutations in the high-grade component supporting molecular evolution. The high prevalence of *ERBB2* amplification in these tumors distinguishes them from pure serous and pure endometrioid carcinomas and suggests a potential role for HER2-targeted therapies in this subset of patients. These findings reinforce the importance of molecular subtyping. Identifying how combinations of mutated genes, such as *TP53* and *FBXW7* may impact specific cellular pathways will reveal therapeutic targets in a truly individualized way.

## Figures and Tables

**Table 1:** Clinicopathologic features of mixed endometrioid adenocarcinomas compared to serous and endometrioid endometrial adenocarcinomas.

Parameter	Endometrioid Endometrial Carcinoma (EC)	Mixed Endometrial Carcinoma (MEC)	Serous Endometrial Carcinoma (SC)
**N**	10	9	9
**Age** **Median (Range)**	64 (57–82)	73 (53–78)	68 (59–81)
**Clinical stage** **IA** **IB** **II** **IIIA** **IIIB** **IIIC** **IVA** **IVB**	7 2 0 0 1 0 0 0	4 2 1 0 0 0 2	4 2 0 0 0 2 0 1
**BMI average**	38	30	33
**Disease free survival (median in months)**	48 (5–72)	30 (11–72)	24 (11–60)
**Histologic subtype/gradeI**	Grade 1 (3) Grade 2 (4) Grade 3 (3)	SEC/EEC (8) CEC/EEC (1)	All grade 3/high-grade by definition

**Table 2:** Comparative TP53 Gene Mutations in Pure Serous versus Mixed & Mixed Feature Carcinomas.

Tumor Type		*TP53* Gene Mutations (at Exon NM_000546.5)			
		Inactivating	Possible Gain of Function	Truncating	Unknown
**Mixed & Mixed Feature Carcinomas**	**Low stage disease** **Stages 1,2**	p.Val272Leu (missense) p.Arg273Cys (missense) p.Arg273His (missense)	p.Arg175His (missense)	p.Trp53Ter (nonsense) p.Asn29LysfsTer14 (frameshift insertion) p.Arg306Ter (nonsense)	
	**High-stage disease** **Stages 3,4**	p.Arg249Ser (missense)			
**Serous** **Carcinomas**	**Low stage disease** **Stages 1,2**		p.Arg175His (missense) p.Arg175His (missense)	p.Gln317Ter (nonsense)	
	**High-stage disease** **Stages 3,4**	p.Ser241Phe (missense)		p.Arg306Ter (nonsense) p.Arg342Ter (nonsense)	p.Asp281Glu (missense)

## Data Availability

Our data were internally generated and will be made available upon request.
